# 4-Octyl Itaconate ameliorates diesel exhaust particle-induced oxidative stress in nasal epithelial cells

**DOI:** 10.3389/fimmu.2025.1640499

**Published:** 2025-08-22

**Authors:** Hanna Steppuhn, Katja Hohenberger, Susanne Mittler, Sonja Trump, Christine Carvalho, Manfred Rauh, Andreas B. Wild, Nikolaos G. Papadopoulos, Susetta Finotto

**Affiliations:** ^1^ Department of Molecular Pneumology, Friedrich-Alexander-Universität (FAU) Erlangen-Nürnberg, Universitätsklinikum Erlangen, Erlangen, Germany; ^2^ Department of Laboratory Diagnostics, Children’s Hospital, Friedrich-Alexander- Universität (FAU) Erlangen-Nürnberg, Universitätsklinikum Erlangen, Erlangen, Germany; ^3^ Department of Immune Modulation, Dermatology Clinic, Friedrich-Alexander- Universität (FAU) Erlangen-Nürnberg, Universitätsklinikum Erlangen, Erlangen, Germany; ^4^ Department of Allergy, 2nd Pediatric Clinic, National and Kapodistrian University of Athens, Athens, Greece; ^5^ Centre for Respiratory Medicine and Allergy, University of Manchester, Manchester, United Kingdom; ^6^ Bavarian Cancer Research Center (BZKF), Erlangen, Germany; ^7^ Comprehensive Cancer Center Erlangen-EMN (CCC ER-EMN), Erlangen, Germany; ^8^ Deutsches Zentrum für Immuntherapie (DZI), Erlangen, Germany

**Keywords:** reactive oxygen species, itaconate, particulate matter, respiratory mucosa, RNA-seq analysis

## Abstract

**Background and objective:**

Particulate matters such as diesel exhaust particles induce oxidative stress in cells and thereby have a negative impact on health. The aim of this study was to test whether the membrane-permeable, anti-inflammatory metabolite 4-Octyl Itaconate can counteract the oxidative stress induced by diesel exhaust particles and to analyze the downstream-regulated pathways both in human nasal epithelial cells and PBMCs.

**Methods:**

Human nasal epithelial cells were cultured from nasal swabs, and the response of the cells to diesel exhaust particles either alone or in combination with 4-Octyl Itaconatee was investigated using RNA sequencing, qPCR, and cytokine measurement. The presence of reactive oxygen species in the cells was analyzed using CellROX staining and flow cytometric DCFDA assay.

**Results:**

Diesel exhaust particles caused an upregulation of *CYP1A1* in nasal epithelial cells. The administration of 4-Octyl Itaconate reduced the reactive oxygen species and increased the expression of antioxidant genes regulated by the transcription factor NRF2, which was also confirmed in PBMCs. IL-6 secretion from NEC was elevated by diesel exhaust particles and reduced when 4-Octyl Itaconate was administered.

**Conclusion:**

4-Octyl Itaconate can reduce the diesel-exhaust-particle-induced oxidative damage by the activation of NRF2-regulated antioxidative pathways.

## Introduction

Epidemiological and observational studies have shown that exposure to air pollutants like particulate matter (PM) and especially diesel exhaust particles (DEP) is linked to negative cardiopulmonary effects, airway inflammation, and an increased risk for allergic diseases (1). DEP are one of the most prevalent environmental pollutants, especially in urban areas. The particles belong to the fine (PM_2.5_) and ultrafine particle fractions (PM_0.1_) of PM and can penetrate deep into the respiratory tract (2). They consist of a complex mixture of elemental carbon, metals, and organic compounds, including polycyclic aromatic hydrocarbons (PAHs) and nitro-PAHs, which are known carcinogens (3).

DEP have a direct effect on the airway epithelium by triggering oxidative stress, release of pro-inflammatory cytokines, and cell death (4). The contained PAHs trigger cellular pathways, which lead to an increased production of reactive oxygen species (ROS) via the ligand-activated transcription factor aryl hydrocarbon receptor (AhR) (5). Binding of PAH to the AhR leads to the transcriptional activation of xenobiotic-metabolizing phase 1 and phase 2 enzymes, causing ROS production (4).

This study also investigated the role of the mitochondrial metabolite itaconate in modulating cellular responses to oxidative stress. Itaconate, a derivative of the tricarboxylic acid (TCA) cycle, has gained increasing attention in recent years for its role in the modulation of immune reactions and inflammation. Endogenously, itaconate is produced primarily in activated macrophages and other myeloid cells through aconitate decarboxylase 1 (ACOD1, encoded by *IRG1*). In contrast, epithelial cells typically do not express *IRG1* and are thus considered incapable of synthesizing significant amounts of itaconate. Despite this, exogenous application of membrane-permeable itaconate derivatives, such as 4-Octyl Itaconate (4OI), has been shown to exert potent anti-inflammatory and antioxidant effects also in non-myeloid cells. This indicates that 4OI may mimic certain intracellular functions of endogenously produced itaconate independent of *IRG1* expression (6, 7).

Itaconate itself is highly polar and poorly permeable to cellular membranes. To overcome this limitation *in vitro*, membrane-permeable derivatives such as 4OI or dimethyl itaconate are commonly used and generate comparable biological effects to natural itaconate (6). Interestingly, recent studies have also suggested that glucocorticoids may exert their anti-inflammatory action via the activation of the itaconate pathway, further highlighting its relevance in the regulation of immune responses (8).

While traditional antioxidants like glutathione, vitamin C, and N-acetylcysteine directly scavenge reactive oxygen species (ROS), itaconate works through more nuanced mechanisms that involve transcriptional regulation via antioxidant transcription factors like NRF2. Itaconate represents a next-generation antioxidant that regulates inflammation and metabolism rather than acting purely as a ROS scavenger. In 2018, Mills et al. showed in macrophages that the derivate 4OI significantly activated NRF2 and increased the expression of NRF2 downstream antioxidant and anti-inflammatory genes like *HMOX1* and *NQO1* (9). Mechanistically, itaconate exerts its effects via the alkylation of specific cysteine residues on Kelch-like ECH-associated protein 1 (KEAP1) which is a cytosolic repressor of NRF2. This alkylation prevents KEAP1 from promoting NRF2 degradation, thereby allowing NRF2 to accumulate, translocate to the nucleus, and drive the expression of numerous cytoprotective, anti-oxidative, and anti-inflammatory genes (9). Studies investigating the effects of the derivative 4OI confirmed the described mechanism of NRF2 stabilization and activation of downstream genes *in vitro* and *in vivo*.

The previous research has been mainly focused on the effects of itaconate and 4OI on murine models and macrophages—for example, in Acod1-/- mice, it was demonstrated that the exogenous application of 4OI attenuated the particulate-matter-induced inflammation in macrophages (10). In this paper, we additionally examined its role in human epithelial cells of the upper respiratory tract, thereby shedding light on the body’s point of contact with air pollution such as DEP. The potential protective effects of exogenously supplied 4OI on the cells were investigated to determine whether itaconate is able to ameliorate the inflammation and oxidative stress caused by DEP.

## Methods

### Human study

From February to June 2024, volunteers aged between 22 and 28 years were recruited in Erlangen at the Molecular Pneumology Department in accordance to our study named AZCRA (“Investigation of the role of cytokines, chemokines and their receptors in the inflammatory process in asthma patients”) which was approved by the ethics committee of the FAU Erlangen-Nürnberg, Germany (Reg. No. 20-315_4-B, DRKS-ID: DRKS00023843). Written informed consent was obtained from all participants included in the study. Excluded from participation in the study were subjects suffering from chronic respiratory diseases such as asthma and people on medication that affects the immune system. All participant data were handled in an anonymized manner to ensure confidentiality. Nasal swabs, blood samples, and lung function tests were taken and questionnaires answered. The clinical characteristics of the subjects are displayed in **Tables 1** and **2**, and further results can be found in **Supplementary Figure S3**.

**Table 1:** Summary of demographic and clinical characteristics of study participants (AZCRA).

Demographic or clinical parameter	Allergic	Healthy control
Participants (*n*)	11	8
Age (years)	25.27 ± 2.22	24.63 ± 2.23
Gender (% female)	54.50%	87.50%
BMI (kg/m²)	24.32 ± 3.29	21.36 ± 1.57
Total IgE in serum (kU/L)	110.14 ± 72.90	58.34 ± 73.79
*Staphylococcus aureus* in nasal swab	75%	50%
Spirometry and lung volumes		
FEV_1_/FVC (%)	88.25 ± 4.84	88.46 ± 4.81
FEV_1_% predicted	98 ± 13	91 ± 16
FeNO (ppb)	15.15 ± 10.13	5.64 ± 2.31

Data are presented as *n*, mean ± SD or *n* (%), unless otherwise stated.

BMI, body mass index; FEV1, forced expiratory volume in 1 s; FVC, forced vital capacity; FeNO, fractional exhaled nitric oxide in parts per billion.

**Table 2:** Characterization of the AZCRA cohort.

Subject ID	NEC used for RNA-seq	Cohort	Gender	Age	Allergy	Place of living
580		A	f	23	Grass, birch, rye, artemisia	Suburb, with many green areas
581		A	f	23	Grass, rye	Suburb, with few green areas
582		A	m	22	Grass, rye, house dust mite	Suburb, with few green areas
586		A	f	24	grass, house dust mite, rye, birch, animal hair	Suburb, with few green areas
587	Yes	A	f	26	Grass, rye	Suburb, with few green areas
588		A	m	23	Grass, artemisia, rye, birch, ambrosia	Suburb, with few green areas
591	Yes	A	m	26	House dust mite, grass, rye	Suburb, with many green areas
592		A	m	28	Grass, rye	urban
594	Yes	A	f	28	Grass, birch	Suburb, with many green areas
595		A	m	27	Grass, birch, house dust mite, wasp	rural
596		A	f	28	house dust mite, animal hair	Suburb, with many green areas
583	Yes	C	f	22	/	Suburb, with few green areas
584	Yes	C	f	22	/	Suburb, with many green areas
585	Yes	C	f	24	/	Suburb, with few green areas
589		C	f	22	/	Suburb, with few green areas
590		C	f	26	/	Suburb, with few green areas
593		C	f	27	/	urban
597		C	m	27	/	Suburb, with few green areas

Allergy based on previous prick test and RAST.

A, allergic; C, healthy control.

### Culture and stimulation of NEC

Nasal epithelial cells (NEC) were collected from the subjects via nasal swabs. The cells were cultured in PneumaCult™-Ex Plus Medium (Stemcell Technologies) as previously described (11) with equal parts of Pneumacult + and Pneumacult ++ in a humidified incubator at 37 °C. The NEC were seeded in 12-well plates pre-coated with Collagen-R (SERVA Electrophoresis GmbH). As required, the cells were split, and new medium was added. After reaching 70%–80% confluence, the cells (*n* = 6) were exposed to the following substances for 24 h: DEP SRM 2975 (CAS-number: 1333-86-4, Sigma-Aldrich) was mixed with PBS to the desired concentration and homogenized in a heated ultrasonic bath. Before each subsequent use, the vial was vortexed for several minutes. Unless otherwise stated, a concentration of 50 µg/mL was used in the experiments. The concentration was selected based on previously published data (10, 20, 21) and preliminary experiments that assessed cell morphology, growth behavior, and microscopic viability at different DEP concentrations.

4-Octyl itaconate (Biomol GmbH) was mixed with DMSO to a stock with 50 mM, and a concentration of 50 µM 4OI (=12 µg/mL) was used for the following experiments. The concentration of 4OI used was selected based on previously published data (9, 22).

### Measurement of oxidative stress

NEC was transferred to collagen-coated chamber slides, and the adherent cells were exposed to DEP 50 µg/mL, 4-octyl itaconate 50 µM, and their combination for 24 h. For assessment of oxidative stress, the cells were incubated with 5 μM CellROX™ Green Reagent (Thermo Fisher) for 30 min, protected from light. The fluorescent dye is activated by ROS, binds to the nucleus, and can be detected as a green signal in fluorescence microscopy. The cells were then washed twice with PBS, and images were captured with ×63 magnification using the fluorescence microscope Axio Observer D1 (Carl Zeiss Microscopy). Fluorescence intensity was quantified with ImageJ in two to three randomly selected fields by measurement of the mean fluorescence intensity of the nuclei with subtraction of the background intensity. The intensity of the fluorescence correlates directly with the amount of ROS in the cells and thus enables an assessment of oxidative stress.

### DCFDA assay

Intracellular reactive oxygen species (ROS) levels in NEC were measured using 2’,7’-dichlorodihydrofluorescein diacetate (DCFDA) for flow cytometry (Sigma-Aldrich, cat. no. D6883). The cells were cultured under standard conditions until ~80% confluence and then subjected to the respective experimental conditions. As positive control for ROS detection, additional cells were treated with 100 µM hydrogen peroxide (Roth) for 30 min at 37 °C. Following each treatment, the cells were washed twice with PBS and incubated with 10 µM DCFDA in serum-free medium for 30 min at 37 °C in the dark. After staining, the cells were washed again with PBS to remove excess dye. The cells were detached using trypsin-EDTA, collected by centrifugation (1,500 rpm, 5 min, 4°C), and resuspended in FACS buffer. Fluorescence was immediately measured using a FACSymphony A1 flow cytometer (BD). A minimum of 40,000 events were acquired per sample. Data were analyzed using Kaluza Analysis Software (Beckman Coulter), and ROS levels were quantified as median fluorescence intensity (MFI). The gating strategy used is shown in **Supplementary Figure S1**.

### Measurement of cytokines

Cytokine concentrations in the samples were quantified via multiplex ELISA using the LEGENDplex™ Human Inflammation Panel 13-plex (Biolegend). The assay was performed with an adapted protocol using 5 µL of the sample. The samples were mixed with 5 µL assay buffer, 5 µL beads, and 5 µL detection antibody and incubated for 2 h in the dark on a shaker. Then, 5 µL SA-PE was added and incubated for a further 30 min while shaking. The supernatant was then washed with 150 µL 1× wash buffer, and after centrifugation, the supernatant was removed by inverting the plate. After resuspension of the beads in 1× wash buffer, the cytokine levels were measured using the FACSymphony A1 flow cytometer (BD). Data analysis was performed using Kaluza Flow Cytometry Software v2.3 (Beckman Coulter). Cytokine levels were determined based on the median fluorescence intensity (MFI), which was normalized to standard curves run in parallel on each assay plate.

### TGF-β1 ELISA

A human TGF-β1 ELISA (cat. no. DY240-05, R&D systems) using cell culture supernatants from stimulated PBMCs was used to quantify the released TGF-β1 levels. The assay was conducted according to the manufacturer’s protocol. Supernatants were collected after stimulation and stored at −80 °C until analysis. Prior to measurement, the samples were activated by acidification via 1 N HCl and, after 10 min, neutralized with an equal volume of 1.2 N NaOH/0.5 M HEPES. Activated samples and standards (diluted in 1% BSA in PBS) were added in duplicate to previously coated plates with capture antibody and incubated for 2 h at room temperature. After washing, detection antibody was added for 2 h, followed by incubation with streptavidin-HRP and subsequent substrate development. The reaction was stopped after 20 min with H_2_SO_4_, and absorbance was measured using a microplate reader.

### RNA sequencing of NEC

Total RNA was extracted from NEC using the RNeasy Mini Kit (Qiagen) according to the manufacturer’s instructions. Further quality control and sequencing were carried out by Novogene (Munich, Germany). HISAT2 was used to map the clean reads to the human reference genome GRCh38/hg38.

### Annexin V/PI staining

Annexin V/PI staining in flow cytometry was performed according to the manufacturer’s instructions. NEC were harvested with trypsin-EDTA and resuspended in 50 µL 1× binding buffer containing 2.5 µL annexin V-APC (BD) and 2.5 µL propidium iodide (BD). After an incubation time of 15 min at room temperature, FACS buffer was added to the cells. For proper gating, single-stained controls (annexin V only and PI only) as well as unstained cells were included in each experiment. Data acquisition was performed using the FACSymphony A1 flow cytometer (BD), and data analysis was performed using Kaluza Flow Cytometry Software v2.3 (Beckman Coulter). The gating strategy used is shown in **Supplementary Figure S2**.

Additional details on the additional methods and primers used are provided in the **Supplementary Material**.

### Statistical analysis

Statistical analysis and graphic illustration were performed with GraphPad Prism version 10 (GraphPad Software). All datasets were analyzed for normal distribution before statistical analysis was performed. Statistical significance was calculated using two‐tailed Student’s *t*-test for the analysis of two-group comparisons and one-way ANOVA for multiple comparisons. Significances are shown as *p ≤ 0.05 and **p ≤ 0.01.

## Results

### Effect of diesel exhaust particles on nasal epithelial cells

With regard to environmental pollution as a negative factor on health, the aim of this study was to investigate how DEP affect the nasal mucosa. For this objective, swabs were taken from the participants, and NEC were cultured from these in PneumaCult™-Ex Plus medium, which is a special medium for the expansion of human airway epithelial cells. A time-lapse video of the proliferating cells within 24 h is provided online (**Supplementary Video S1**). This shows the typical growth behavior of NEC without additional substances such as DEP.

**Figure 1:**
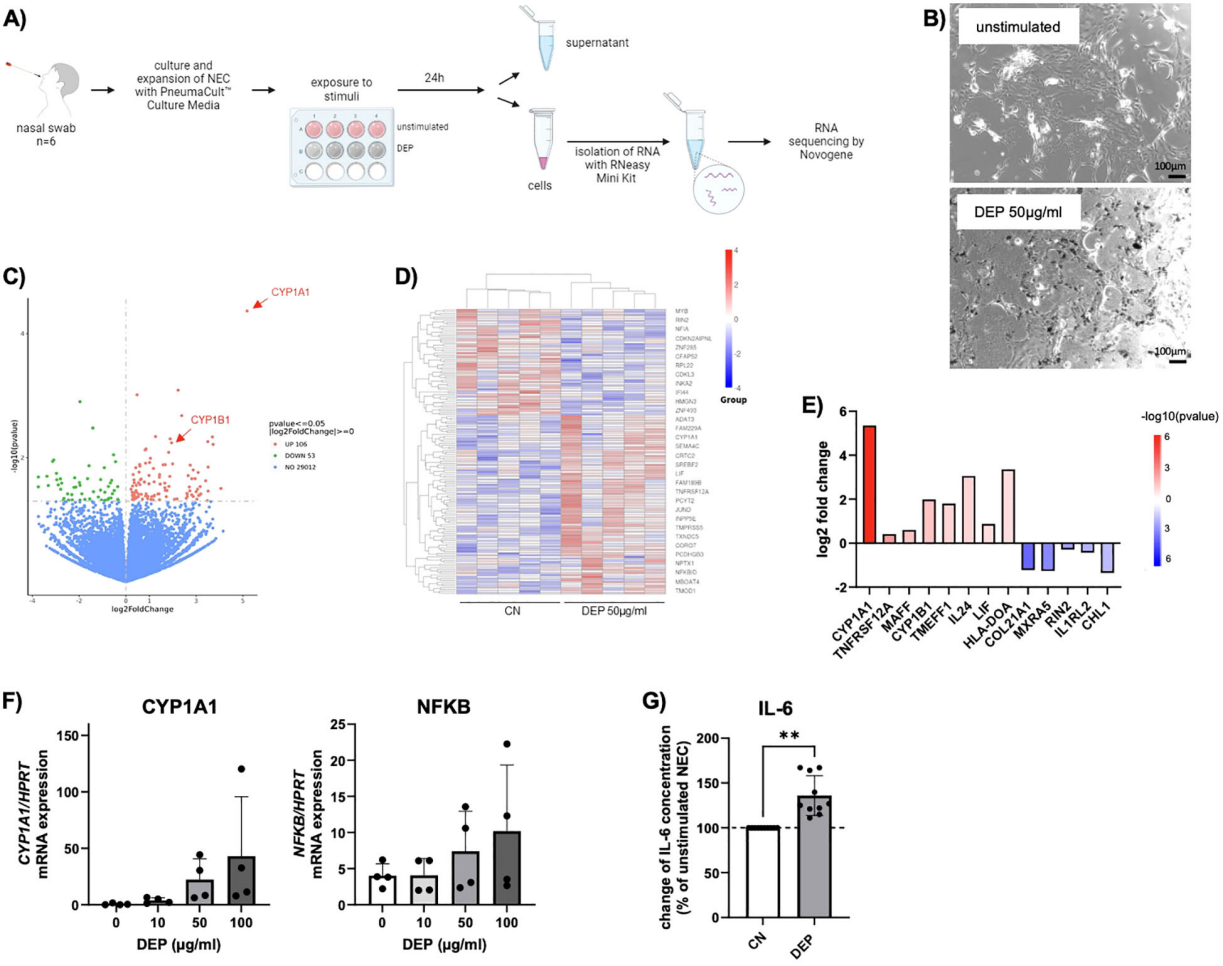
*Effect of diesel exhaust particles (DEP) on nasal epithelial cells (NEC).* **(A)** Experimental design of NEC culture: Nasal swabs were taken from the participants and cells were cultured. After reaching confluence, cells of 6 subjects were exposed to DEP or treated with medium only as control. After 24h supernatant was collected and RNA of the cells isolated, from which RNA sequencing was performed. **(B)** Representative pictures of NEC in absence and presence of 50µg/mL DEP (Nist2975). Scale bar: 100µm. **(C-E)** RNA sequencing data for NEC treated with DEP (50µg/mL) for 24h and unstimulated control (n=6). Data are presented as a heat map illustrating differential gene expression **(D)** and a volcano blot **(C)**. Red datapoints represent genes significantly upregulated. Green datapoints represent genes significantly downregulated with p<0,05. **(E)** Quantification (log2fold change) of differentially expressed genes. **(F)** qPCR analysis of CYP1A1 and NFKB mRNA expression of NEC stimulated for 24h with different DEP concentrations (0-100µg/mL) (n=4). **(G)** IL-6 levels were measured via LEGENDplex in the supernatant of DEP 50µg/mL NEC culture and calculated in relation to unstimulated NEC cells (n=10). N values are given per group. Bar charts indicate mean values +/- SD using paired t-Test **(G)**. **p ≤ 0.01.

Then, 50 µg/mL DEP was added to the medium of the NEC and analyzed to determine the effects on these cells (**Figures 1A, B**). Using RNA sequencing, it was investigated which genes and pathways are activated or downregulated in these cells. It was found that particularly the gene *CYP1A1* (*cytochrome P450 family 1 subfamily A member 1*) was strongly upregulated in cells exposed to DEP for 24 h compared to unstimulated cells (**Figures 1C–E**). The gene *CYP1B1*, which is structurally similar to *CYP1A1*, was also found to be more highly expressed. Both are tightly linked to the formation of ROS (6). A targeted analysis of the RNA-seq data on other CYP enzymes did not detect further members of the family as being more strongly expressed upon DEP exposure. In subsequent studies, a dose–response relationship between *CYP1A1* expression and DEP concentration was shown (**Figure 1F**). Similarly, the expression of NF-κB, a transcription factor critically involved in the regulation of apoptosis, also showed a trend toward upregulation with increasing DEP exposure. However, due to the limited sample size, these observed increases in expression did not reach statistical significance for either gene (**Figure 1F**). In addition, the released cytokines were measured using multiplex ELISA. This analysis showed that the DEP-exposed NEC secreted significant amounts of the pro-inflammatory cytokine IL-6 into the supernatant compared to unstimulated NEC (**Figure 1G**).

### 4-Octyl Itaconate decreased both ROS formation and IL-6 induced by DEP in nasal epithelial cells

**Figure 2:**
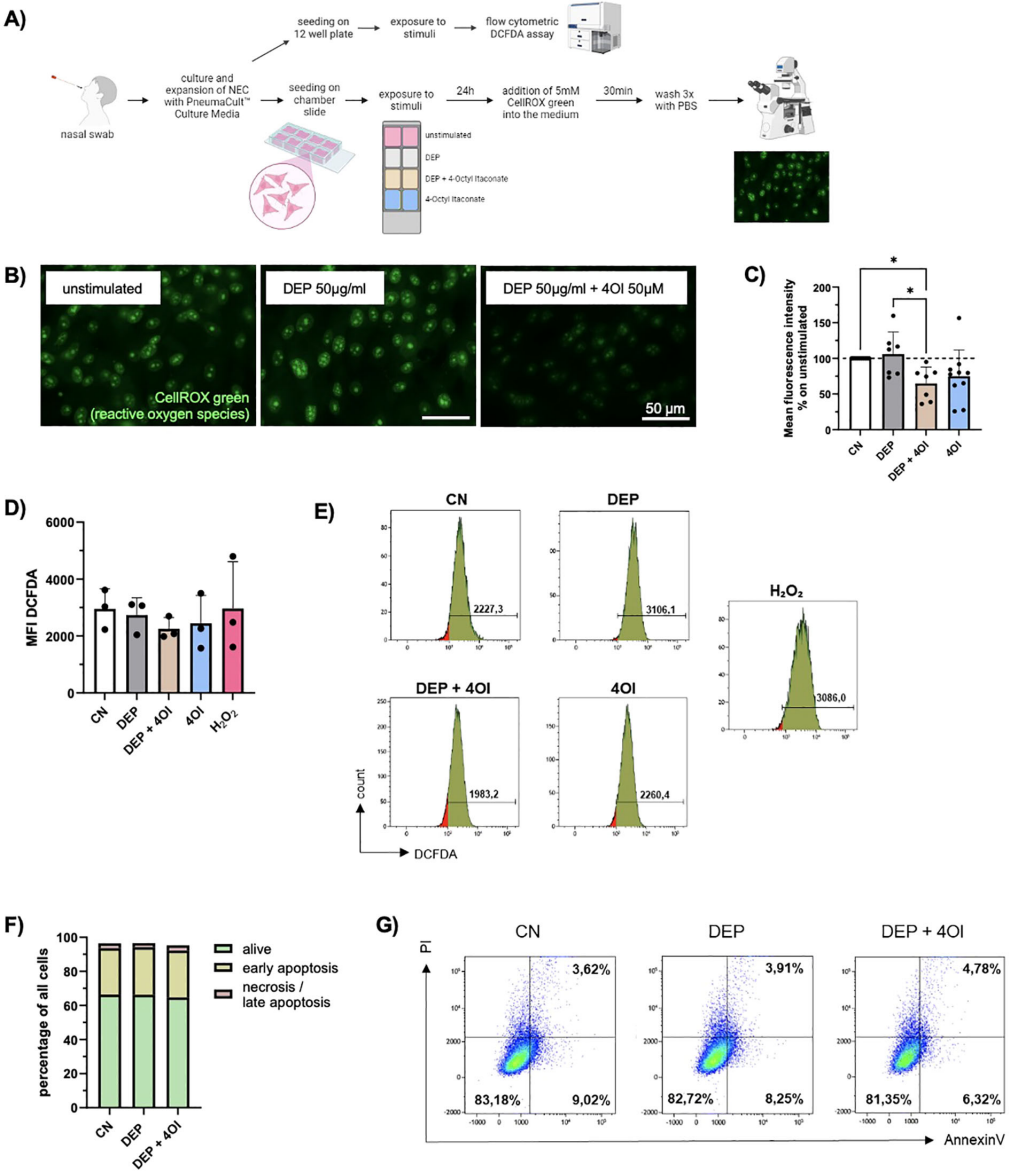
*DEP induce ROS formation which is ameliorated by addition of 4-Octyl Itaconate (4OI*). **(A)** Experimental design: Nasal swabs were taken, and cells were cultured. When a sufficient number of cells was reached, the cells were plated on collagen-coated chamber slides and stimulated with DEP +/- 4OI and 4OI alone for 24h. The ROS were then visualized using the fluorescence staining CellROX Green. In addition, exposed cells were analyzed via flow cytometric DCFDA assay for quantitative assessment of ROS. **(B)** Images of NEC after staining with CellROX Green. Pictures were taken with the Axio Observer D1 microscope (63x magnification). Scale bar: 50µm. **(C)** Mean fluorescence intensity of the nuclei normalized to the respective control (n=7-10). **(D)** Mean DCFDA fluorescence intensity in NEC analyzed with DCFDA assay (n=4). **(E)** Representative histograms of the respective conditions and H_2_O_2_ as positive control for DCFDA assay. **(F)** Evaluation of cell viability via Annexin V/PI staining. The amount (%) of living cells (green), early apoptotic cells (yellow) and necrotic/late apoptotic (red) in relation to total cells were calculated (n=4). **(G)** Representative flow cytometry dot plots showing NEC of one representative subject stimulated with DEP +/- 4OI. Viable cells are Annexin V^-^/PI^-^ (lower left quadrant), early apoptotic cells are Annexin V^+^/PI^-^ (lower right quadrant), late apoptotic/necrotic cells are Annexin V^+^/PI^+^ (upper right quadrant). N values are given per group. Bar charts indicate mean values +/- SD using one-way ANOVA **(C)**. *p ≤ 0.05.

Since particularly the expression of *CYP1A1*, which is associated with the production of ROS, was increased in the RNA-seq of DEP-exposed NEC, a subsequent analysis focused on oxidative stress. For this reason, the oxidative imbalance in NEC was analyzed and quantified by detection of ROS via CellROX staining and DCFDA assay (**Figure 2A**). The intensity of the fluorescence correlates directly with the amount of ROS in the cells and thus enables an assessment of oxidative stress. In NEC exposed to DEP, increased fluorescence intensity of CellROX was observed, indicating the increased formation of ROS (**Figure 2B**).

Considering the oxidative imbalance in these cells, the next step was to explore substances that would help the cells to deal with stress. Therefore, the effect of the metabolite 4OI on the cells was investigated as it has shown anti-inflammatory and anti-oxidative effects. An analysis of the ROS present in NEC using CellROX staining showed a significantly reduced fluorescence intensity when 4OI is added to DEP-exposed cells (**Figures 2B, C**). Since the cells of different test subjects had varying baseline levels of ROS in the unstimulated samples, the other measured values were normalized to the respective control.

To better quantify intracellular ROS, a flow-cytometry-based DCFDA assay was performed. NEC were exposed for 24 h to the same concentrations of DEP, 4OI, and their combination as used in the previous experiments. The DCFDA (2′,7′-dichlorofluorescin diacetate) assay detects ROS by utilizing a cell-permeable, non-fluorescent dye that is deacetylated by intracellular esterases and subsequently oxidized by ROS to form the highly fluorescent compound DCF. The resulting fluorescence intensity directly reflects the intracellular ROS levels and was measured by flow cytometry. Hydrogen peroxide (H_2_O_2_) was used as a positive control and yielded the strongest fluorescence signal, confirming the sensitivity and reliability of the assay. Although no statistically significant changes were observed, treatment with 4OI decreased the ROS levels in NEC (**Figures 2D, E**).

Furthermore, it was investigated whether there is an effect of DEP and 4OI on cell survival. Using annexin V/PI staining, no significant changes in cell viability, apoptosis, or necrosis were observed upon exposure (**Figures 2F, G**).

### 4-Octyl Itaconate activates the transcription of NRF2-regulated genes in DEP-exposed NEC and PBMCs

**Figure 3:**
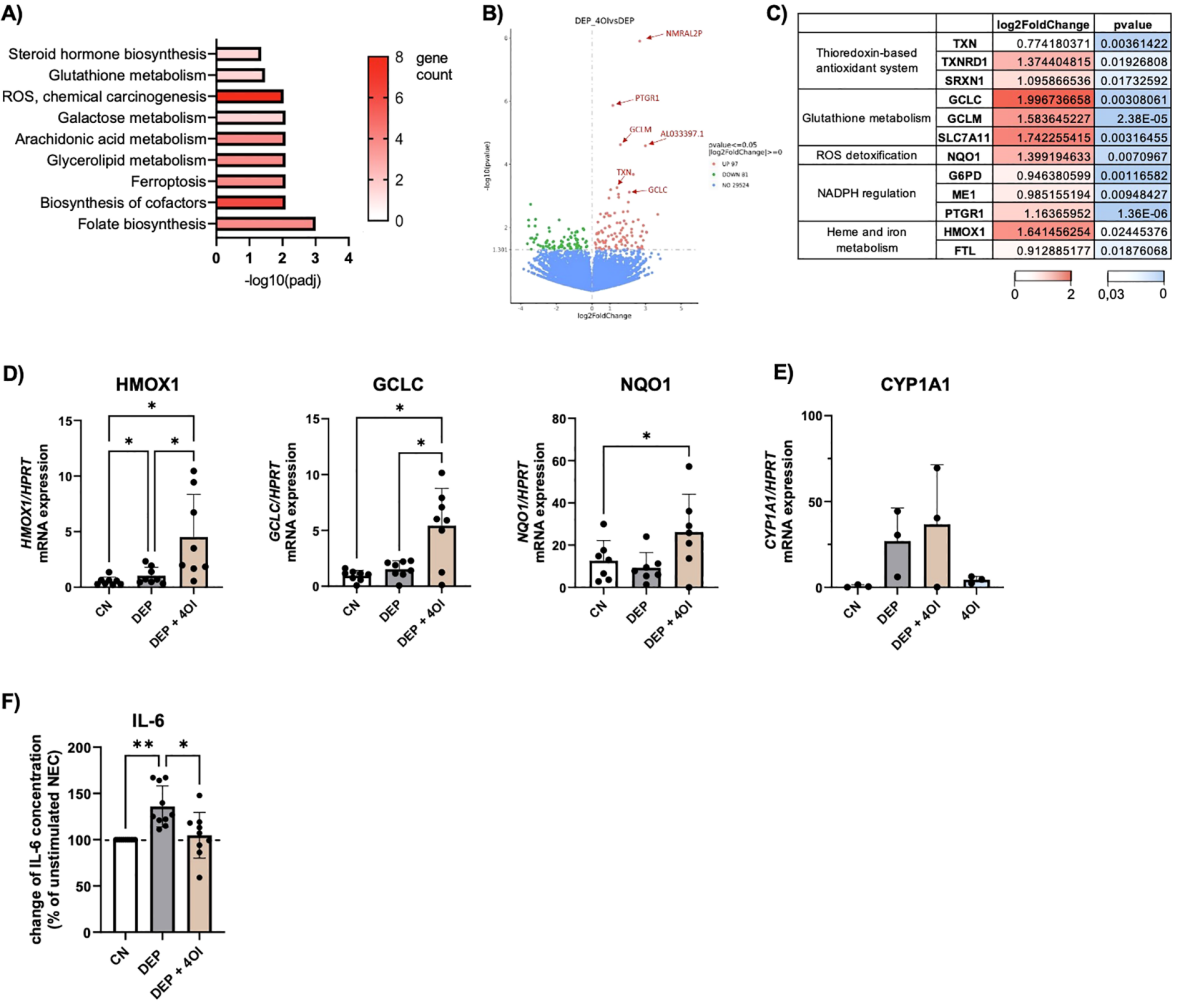
*4-Octyl Itaconate activates NRF2 regulated genes in NEC*. **(A-C)** Results of the RNA-seq: DEP 50µg/mL stimulated NEC were compared to NEC stimulated with 50µg/mL DEP + 50µM 4-Octyl Itaconate (4OI) (n=6). **(A)** KEGG pathway analysis of genes with p ≤ 0,05. **(B)** Volcano plot of differentially expressed genes. Red datapoints represent genes significantly upregulated in DEP–4OI-co-stimulated samples. Green datapoints represent genes significantly downregulated. **(C)** Tabular representation of various genes that are known as target genes of NRF2, with information on the log2 fold change and p value color-coded. **(D)** mRNA expression of HMOX1, GCLC, NQO1 in relation to HPRT was measured via qPCR (n=7-8). **(E)** mRNA expression of CYP1A1 in relation to HPRT (n=3). **(F)** IL-6 levels were measured with LEGENDplex in the supernatant of DEP 50µg/mL +/- 50µM 4OI NEC culture and calculated in relation to unstimulated NEC (n=10). N values are given per group. Bar charts indicate mean values +/- SD using one-way ANOVA **(D, F)**. *p ≤ 0.05; **p ≤ 0.01.

To understand which changes occur at the transcriptional level, RNA sequencing was performed in NEC treated with DEP alone or with additional 4OI. The pathway analysis (**Figure 3A**) showed that pathways associated with glutathione metabolism and ROS were upregulated by the addition of 4OI on top of DEP. A closer examination of the differentially expressed genes revealed that most of the genes were regulated by the transcription factor NRF2. An overview of key genes and their functional roles is presented in **Figure 3C**, where log2 fold changes and *p*-values from the experiment are also provided. It illustrates that NRF2-associated genes were upregulated following treatment with 4OI, which strongly supports the activation of NRF2. Morgenstern et al. (12) identified *GCLC*, *GCLM*, *HMOX1*, *NQO1*, *SRXN1*, and *TXNRD1* as a robust panel of NRF2 target genes. Each of these genes was significantly upregulated in our dataset, which leads to the assumption of NRF2 activation in response to 4OI treatment. No differences were found between cells from healthy or allergic participants.

Additionally, a qPCR analysis of NEC was performed with a larger number of samples to further analyze the NRF2-targeted genes (**Figure 3D**). This confirmed the higher expression of *HMOX1*, *GCLC*, and *NQO1* in DEP–4OI-co-stimulated NEC, supporting the finding of NRF2 induction by 4OI. When the cells were stimulated with DEP alone, there was no change in gene expression in NRF2-regulated genes. However, a significant influence of 4OI on *CYP1A1*, which was upregulated by DEP, was not detected (**Figure 3E**). It can therefore be concluded that 4OI primarily activates NRF2 signaling but does not interfere with the upstream induction of *CYP1A1* by DEP.

A cytokine analysis of the NEC supernatants revealed that the elevated IL-6 release induced by DEP exposure (**Figure 1G**) was reduced to baseline levels following co-treatment with 4OI. In some cases, IL-6 levels were even suppressed below baseline concentrations (**Figure 3F**).

**Figure 4:**
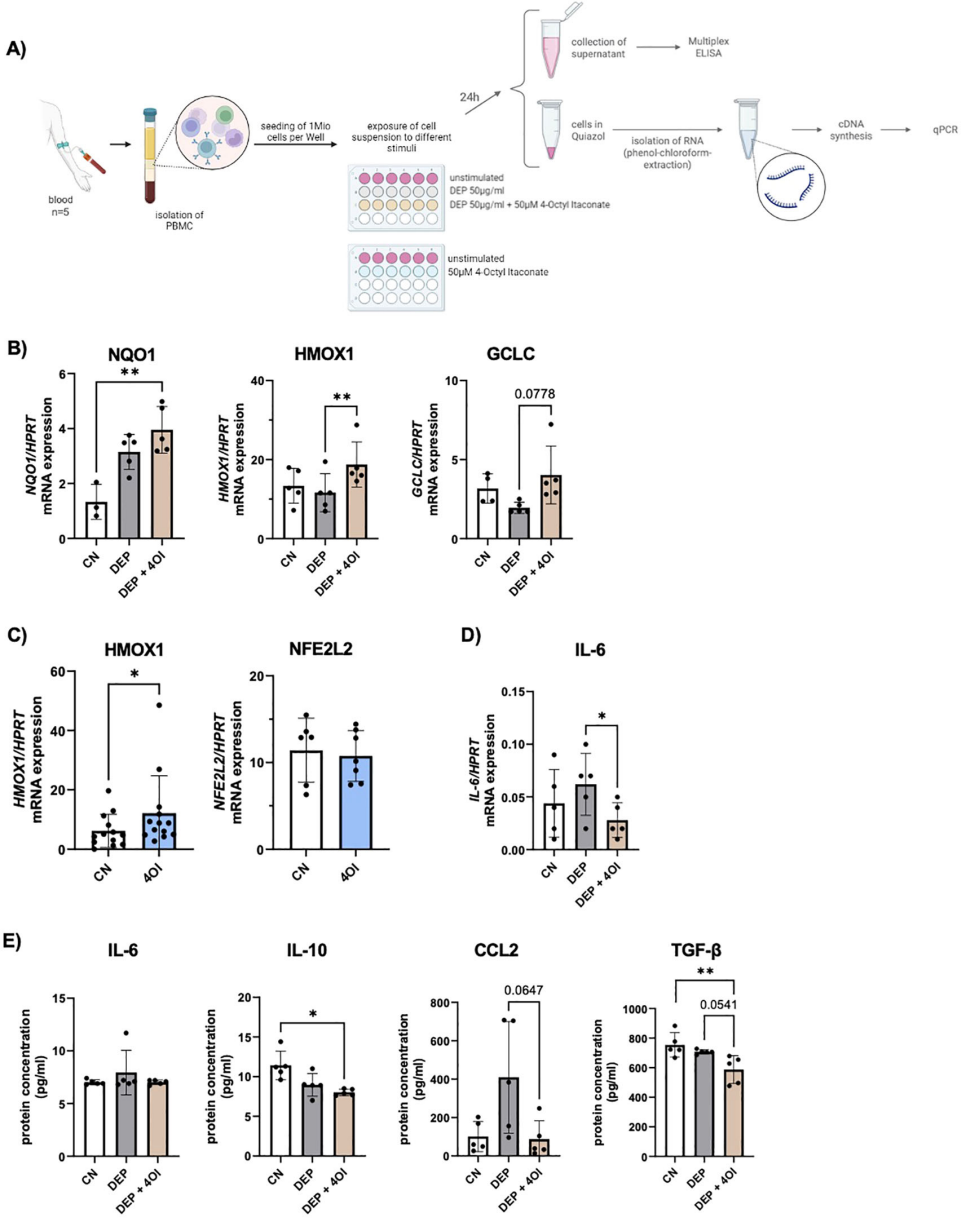
*Effect of diesel exhaust particles (DEP) and 4-Octyl Itaconate (4OI) on human PBMCs.* **(A)** Experimental design: After isolation of PBMCs, the cells were exposed to the following stimuli for 24h: DEP 50µg/mL, 50µM 4OI, and the combination of both, as well as unstimulated cells. After 24h, the supernatant was removed for subsequent cytokine measurement, and the RNA was isolated. **(B)** mRNA expression of NQO1, HMOX1 and GCLC in relation to HPRT was measured by qPCR (n=5). **(C)** mRNA expression of HMOX1 (n=13) and NFE2L2 (n=7) in relation to HPRT was measured by qPCR in PBMCs stimulated with 4OI. **(D)** mRNA expression of IL-6 in relation to HPRT was measured by qPCR (n=5). **(E)** Cytokine levels were measured with multiplex ELISA (LEGENDplex) in the cell culture medium of PBMCs exposed to DEP 50µg/mL +/- 50µM 4OI (n=5). N values are given per group. Bar charts indicate mean values +/- SD using one-way ANOVA **(B, D, E)** and paired t-Test **(C)**. *p ≤ 0.05; **p ≤ 0.01.

To check whether the same effects also occur in other cell types, blood was taken from the participants, and PBMCs were isolated. Cultured cells were exposed to 50 µg/mL DEP, 50 µM 4OI, and a combination of both, which are the same conditions as those used for the experiments with NEC. After 24 h, the cells were harvested, the RNA of the cells was isolated, and cytokines in the cell culture medium were measured via multiplex ELISA (**Figure 4A**).

Subsequent mRNA analysis by qPCR showed an upregulation of *NQO1*, *HMOX*1, and *GCLC* in PBMCs that were additionally treated with 4OI (**Figure 4B**). These are NRF2-targeted genes that were also activated in the NEC exposed to the respective conditions. Without exposure to DEP, the PBMCs showed a significant increase in *HMOX1* expression when stimulated with 4OI for 24 h (**Figure 4C**). A comparison of RNA from samples exposed to 4OI compared with unstimulated cells showed no change in *NFE2L2* expression, which is the gene encoding for the transcription factor NRF2 (**Figure 4C**). It can therefore be assumed that the activation of NRF2 by 4OI does not occur via transcription activation but via other mechanisms such as modulation of the regulator KEAP1.

Furthermore, gene expression analysis revealed a reduced IL-6 mRNA expression due to 4OI addition to DEP-treated PBMCs (**Figure 4D**), which is consistent with the reduced IL-6 levels observed in the supernatants of stimulated NEC (**Figure 3F**). In contrast to these results at the transcriptional level, an analysis of secreted cytokines via multiplex ELISA did not show a change in IL-6 concentration upon treatment (**Figure 4E**). To further compare the cytokine secretion profiles between PBMCs and NEC, additional multiplex analyses were conducted. A significant reduction in IL-10 secretion was observed in DEP–4OI-co-stimulated PBMCs compared to unstimulated controls, and elevated levels of CCL2 in DEP-treated NEC were reduced back to the baseline level by 4OI (**Figure 4E**). In contrast, the other pro-inflammatory cytokines such as TNF-α, IL-1β, and IL-18 remained unchanged across the different treatment conditions (**Supplementary Figure S4**).

In addition, an ELISA was performed to quantify activated TGF-β1 in the PBMC medium. The results demonstrated a significant reduction in TGF-β1 levels following 4OI treatment (**Figure 4E**). Given TGF-β’s role as a key immunoregulatory cytokine, often upregulated in response to oxidative stress and involved in promoting tissue remodeling and fibrosis, its suppression by 4OI may indicate a modulatory effect on oxidative-stress-related signaling pathways and downstream immunosuppressive responses.

## Discussion

In this study, we first investigated the effects of DEP on NEC and examined whether they induce oxidative stress as shown in previous studies (4, 5, 13). Our RNA sequencing data confirmed the induction of *CYP1A1* and *CYP1B1* after DEP exposure, which, alongside other *in vitro* studies, was recently shown in an *in vivo* study in humans by Friberg et al. (14). The increased expression of these genes provides an explanatory approach as to how DEP cause an oxidative imbalance in the cells. Additionally, we visualized the presence of intracellular ROS in NEC using CellROX Green staining and have quantified ROS with a flow cytometric DCFDA assay. Based on this, we searched for ways to restore the oxidative balance in the cells, whereupon we investigated 4OI, which is a membrane-permeable derivative of itaconate. Upon 4OI treatment, RNA sequencing of NEC revealed an activation of pathways associated with glutathione and ROS, and a closer analysis showed that particularly genes regulated by the transcription factor NRF2 were more highly expressed. Based on the panel described by Morgenstern et al. (12), the upregulation of this NRF2 target genes clearly indicates NRF2 activation in response to 4OI treatment. The protective effect of 4OI observed in this study opens new avenues to ameliorate the harmful impact of DEP. By activating the NRF2 pathway, 4OI enhanced the expression of antioxidant enzymes and reduced the ROS. The associated pathways and anti-inflammatory effects of itaconate were summarized in a review by Shi et al. (15).

In this study, no measurable induction of *NFE2L2* expression was observed in PBMCs following 4OI exposure despite the clear upregulation of several NRF2-regulated target genes. While other studies have reported a mild transcriptional upregulation of *NFE2L2* in response to NRF2 activation (16), our findings align with the notion that functional NRF2 signaling can occur independently of increased *NFE2L2* transcription. This supports the model in which 4OI activates NRF2 primarily through the modulation of the regulator KEAP1 rather than through the enhanced transcription of the *NFE2L2* gene itself (9).

Additionally, we observed an inhibitory effect of 4OI on the secretion of the pro-inflammatory cytokine IL-6 in NEC, highlighting a broader anti-inflammatory role of this metabolite. Similarly, DEP–4OI-co-stimulated PBMCs showed a significant reduction in IL-6 mRNA expression, but no change in the secretion of this cytokine could be detected at the protein level, suggesting potential post-transcriptional regulation or differences in secretion dynamics. In conclusion, it can therefore be said that 4OI also exerts its anti-inflammatory effects via limiting the release of IL-6 in NEC but not in PBMCs. Furthermore, Sohail et al. (18) report a significant downregulation of TNF-α, IL-6, and IFN-β in PBMCs following 4OI treatment, which were not present in our model. In contrast, our cytokine analysis via multiplex ELISA showed that exposure to DEP elevates CCL2 secretion in PBMCs, a response that is reversed by co-treatment with 4OI, returning the CCL2 levels to those observed in unstimulated controls. CCL2, a chemokine involved in monocyte recruitment, is known to be elevated during oxidative stress (23), and we were able to show that this effect can be counteracted by 4OI.

The distinct responses observed in NEC and PBMCs highlight the importance of analyzing multiple cell types to gain a broader understanding of the effects of 4OI. By employing this human dual-cell model, our study integrates both the epithelial interface where NEC serve as the initial barrier to inhaled pollutants as well as the systemic immune context represented by PBMCs. This approach provides a more comprehensive understanding of NRF2-mediated antioxidant and immunomodulatory mechanisms activated by 4OI across different biological compartments.

From a public health perspective, our findings suggest the potential for developing therapeutic strategies using itaconate derivatives to counteract the effects of air pollution on the respiratory tract. Such strategies could be particularly beneficial for urban populations that experience high levels of DEP exposure due to traffic and industrial emissions (4). The application of agents like itaconate could help vulnerable groups to alleviate oxidative stress and reduce inflammation.

Itaconate is also interesting in a clinical context, as a recent study by Auger et al. showed that glucocorticoids exert their anti-inflammatory effect via the increased and sustained endogenous production of itaconate (8). From this, it can be speculated whether an increase in the body’s own itaconate production or a therapeutic administration of the metabolite could be a way to avoid side effects of glucocorticoid therapy in the future.

Despite the insights provided by this study, several limitations should be acknowledged. First, we did not measure NRF2 activity directly. We relied instead on the changes in the expression of NRF2 target genes as described by Morgenstern C et al. (12). While this approach offers valuable insights into the downstream pathways, the direct measurement of NRF2 activity, for example by KEAP1 interaction or western blot, could provide a more comprehensive understanding and strengthen mechanistic claims. It would also be interesting to explore additional pathways modulated by DEP and 4OI, including mitochondrial function or inflammasome.

Secondly, our study is largely conducted with NEC and PBMCs. Although this provides important mechanistic insights, such *in vitro* models have some limitations compared to *in vivo* studies. The cellular responses may not fully replicate the complex environment and interactions occurring *in vivo*. Future studies in animal models and clinical trials are needed to validate these findings and assess the therapeutic potential of NRF2 modulation.

Third, measurements of ROS alone do not allow for a direct link of the production specifically to CYP1A1 activity. Further mechanistic experiments would be required to confirm causality. However, previous studies, such as that summarized in the review by Vogel et al. (5), have found a direct link between CYP1A1 and ROS production. Based on these previous findings and the parallel increase of both CYP1A1 expression and ROS levels in our model, we consider a mechanistic link between CYP1A1 and ROS plausible.

In this study, we exclusively investigated the effects of 4OI, without including other derivatives such as dimethyl itaconate, free itaconate, or endogenously produced itaconate. However, earlier studies have shown that these substances have partially different properties due to their slightly different structures (17, 18). To fully discover the cellular mechanisms and therapeutic potential of itaconate signaling, it will be essential in future research to systematically compare the intracellular effects of various itaconate forms and directly measure the intracellular concentrations of the respective substances.

Finally, in the future, it will also be important not only to look at the effects of individual pollutants but also to investigate their synergistic interactions and effects on human health as suggested in the “SynAir-G” hypothesis (19). Interactions of DEP with other pollutants or allergens may exacerbate adverse health outcomes, further highlighting the need for studies that consider the combined effects of multiple pollutants.

Although limited by the aspects described above, our study provides the basis for protecting the upper respiratory tract from the harmful effects of urban pollutants. Addressing these limitations in future studies will not only strengthen the mechanistic understanding of NRF2 but also advance the potential translation of these findings into therapeutic strategies.

## Data Availability

The raw data supporting the conclusions of this article will be made available by the authors, without undue reservation.
