# Supplementary material for: 4-Octyl Itaconate ameliorates diesel exhaust particle-induced oxidative stress in nasal epithelial cells
Source: Front Immunol. 2025 Aug 22;16:1640499. doi: 10.3389/fimmu.2025.1640499 (PMC12411186; doi:10.3389/fimmu.2025.1640499)
Supplement: Supplementary file 1 [file DataSheet1.pdf]

Supplementary Methodes and Materials

Culture of Nasal Epithelial Cells and stimulation with environmental substances

Nasal epithelial cells (NEC) were collected from the subjects via nasal brushing. The cells were cultured in PneumaCult™-Ex Plus Medium (Stemcell Technologies) with equal parts of Pneumacult + and Pneumacult ++ in a humidified incubator at 37°C with 5% CO_2.

Table S1: Composition of PneumaCult media

PneumaCult Basal Medium	PneumaCult-Ex Plus Basal Medium<br>+ 10% PneumaCult 50X Supplement<br>+ 0,1% Hydrocortisone
PneumaCult +	PneumaCult Basal Medium<br>+ 1% Antibioticum/Antimycoticum<br>+ 0,5% Gentamycin
PneumaCult ++	PneumaCult+<br>+ 5% heat inactivated FCS<br>+ 2% Sodiumbicarbonate

Cells were seeded in 12-well plates pre-coated with Collagen-R (SERVA Electrophoresis GmbH). As required, the cells were split with trypsin-EDTA, and new medium was added. After reaching 70-80% confluence, NEC were exposed to following substances for 24 h.

Conditions:

1. Control (medium only)
2. DEP 50 µg/ml
3. DEP 50 µg/ml + 4-Octyl Itaconate 12µg/ml = 50µM

RNA Isolation and Quantitative Real-Time PCR (qPCR)

Total RNA was isolated from both cultured nasal epithelial cells and stimulated PBMCs using QIAzol® Lysis Reagent (Qiagen) according to the manufacturer's protocol. RNA concentration and purity were determined using a NanoDrop™ spectrophotometer (Thermo Fisher). cDNA was synthesized with the RevertAid First Strand cDNA Synthesis Kit (Thermo Fisher Scientific) according to the manufacturer's protocol. The quantitative real-time PCR was performed with

iTaq Universal SYBR Green Supermix (Bio-Rad Laboratories) using the CFX-96 Real-Time PCR Detection System (Bio-Rad Laboratories). The primers used were ordered from Eurofins Genomics and the respective sequences are shown in Table S2. The gene expression of all analyzed genes was normalized on the housekeeping gene *hHPRT* using the ΔCt method. All reactions were performed in duplicate.

Table S2: qPCR- Primer (5`-> 3`)

Gene	forward	reverse
HMOX-1	CCAGGCAGAGAATGCTGAGTTC	AAGACTGGGCTCTCCTTGTTGC
ACOD1	GTGTTTCACATAGCCAGCCAA	AGTGAATAGCCACACCGTTCA
NFE2L2	CACATCCAGTCAGAAACCAGTGG	GGAATGTCTGCGCCAAAAGCTG
GCLC	GGAAGTGGATGTGGACACCAGA	GCTTGTAGTCAGGATGGTTTGCG
HPRT	TGA CAC TGG CAA AAC AAT GCA	GGT CCT TTT CAC CAG CAA GCT
GPX-4	ACA AGA ACG GCT GCG TGG TGA A	GCC ACA CAC TTG TGG AGC TAG
NQO1	CCTGCCATTCTGAAAGGCTGGT	GTGGTGATGGAAAGCACTGCCT
CYP1A1	GATTGAGCACTGTCAGGAGAAGC	ATGAGGCTCCAGGAGATAGCAG
IL-6	AAA GAG GCA CTG GCA GAA AA	AGC TCT GGC TTG TTC CTC AC

Isolation and culture of PBMCs

Peripheral blood was collected from donors using 9,0ml S-Monovette EDTA tubes (Sarstedt). The blood was diluted with the same volume of room temperature PBS and carefully layered over BioCol with a density of 1.077 g/ml (Bio&SELL). Centrifugation at 800 x g, DEC=0 for 20 minutes at room temperature results in a layer with PBMC according to the density gradient. The mononuclear cell layer was collected and washed with RPMI. Remaining red blood cells were lysed with 5ml ACK lysing buffer (Thermo Fisher) and the collected PBMC were resuspended in RPMI 1640 medium (Anprotec) supplemented with 10% FCS, 1% Penicillin/Streptomycin, and 1% L-glutamine. The number of cells was counted using trypan blue staining in a counting chamber. The isolated PBMCs were seeded in 24-well plates at a concentration of 1 Mio cells/ml and exposed to final concentrations of DEP 50 µg/ml, 4-Octyl

Itaconate 50µM and the combination of both. Cells were incubated at 37°C in a 5% CO_2 atmosphere for 24 h.

Supplementary figure 1

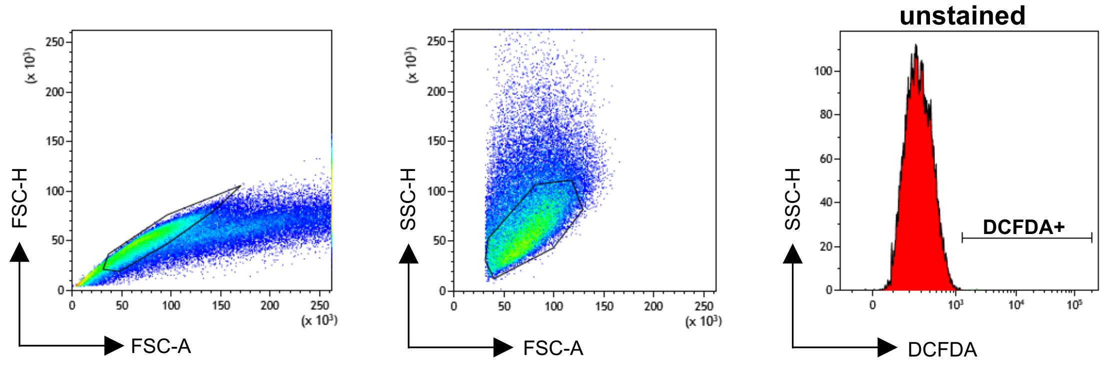

Gating strategy for flow cytometric DCFDA assay on NEC.

Supplementary figure 2

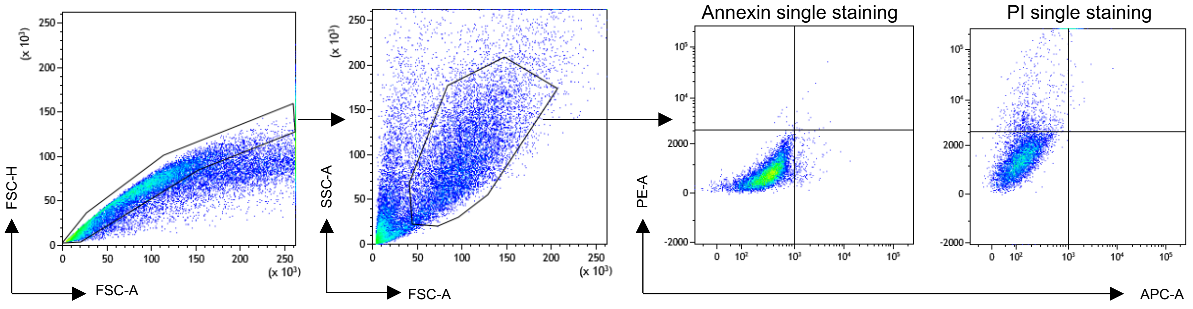

Representative flow cytometry dot plots showing the gating strategy for Annexin V-APC and propidium iodide (PI) staining.

Supplementary figure 3

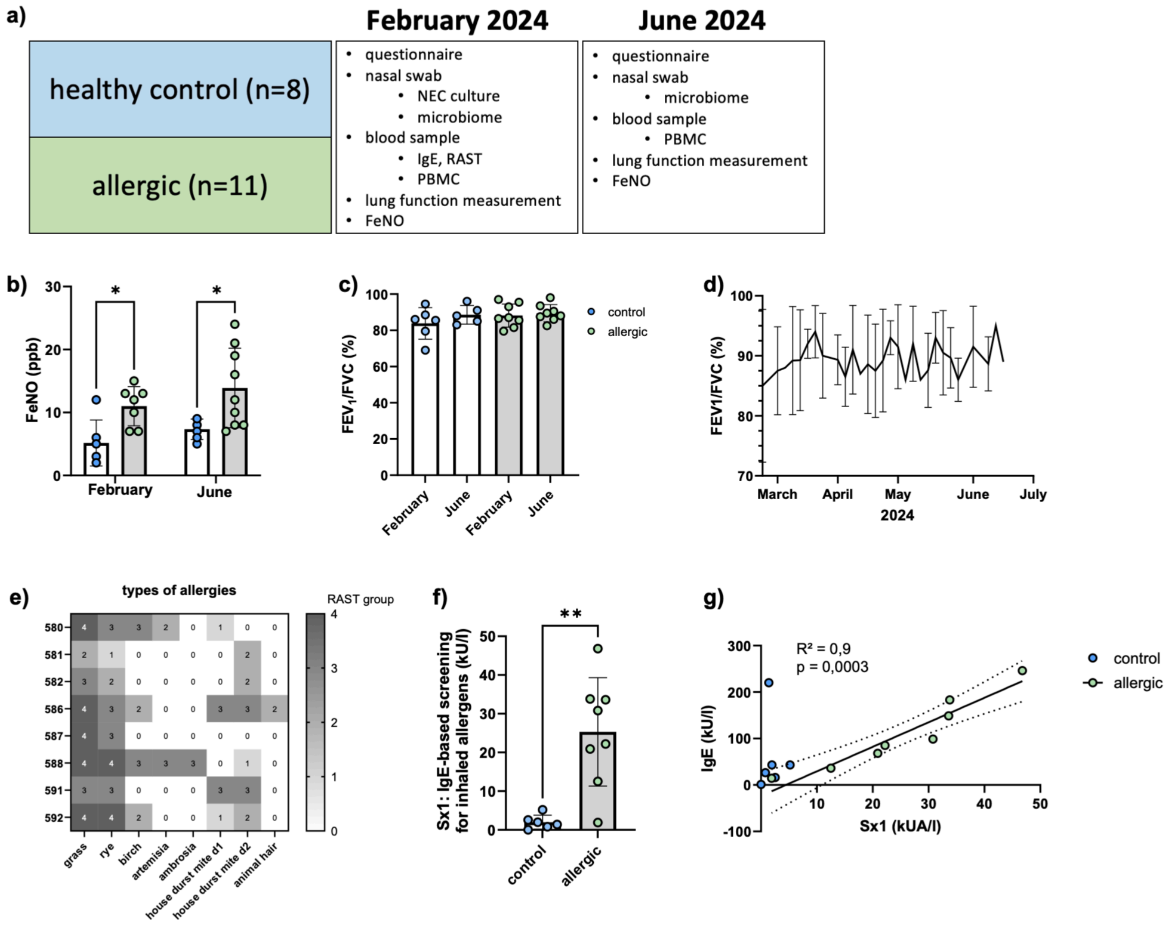

Characteristics of the AZCRA cohort. a) Study design with information on the collected samples and parameters. b) Fractional exhaled nitric oxide (FeNO) in parts per billion, as a parameter for airway inflammation, measured in February and June 2024. c) Measurement of the FEV_1/FVC in February and June 2024. d) Measurement of the FEV_1/FVC in the allergic cohort twice per week at home for four months. e) Representation of specific allergies in the allergic cohort (n=8), based on measurement of specific IgE in the RAST test. f) Results of the screening test for inhaled allergens (Sx1) devided by cohorts ($n_{control}$=6; $n_{allergic}$=8). g) Correlation between total IgE and Sx1 of the allergic subjects, shown as a linear regression with coefficient of determination R^2=0,9 and 95% confidence bands shown. N values are given per group. Bar charts indicate mean values +/- SD using unpaired t-Test (b,f). *p≤0.05, **p≤0.01.

Supplementary figure 4

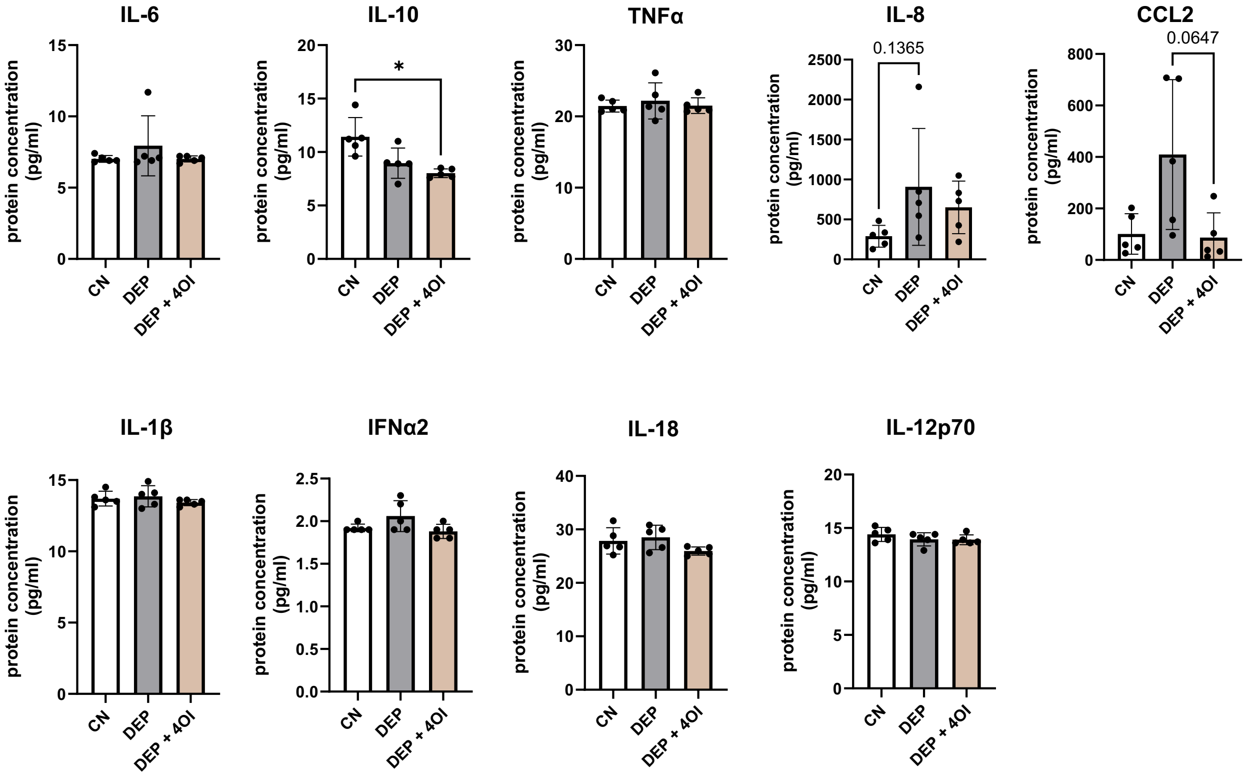

Cytokine levels were measured with multiplex ELISA (LEGENDplex) in the cell culture medium of PBMCs exposed to DEP 50µg/ml +/- 50µM 4OI (n=5). N values are given per group. Bar charts indicate mean values +/- SD using paired t-Test. *p≤0.05
